# Selective Deoxygenation of Biomass Polyols into Diols

**DOI:** 10.3390/molecules30173559

**Published:** 2025-08-30

**Authors:** Juan Carlos Serrano-Ruiz

**Affiliations:** Materials and Sustainability Group, Department of Engineering, Universidad Loyola Andalucía, Avda. de las Universidades s/n, Dos Hermanas, 41704 Seville, Spain; jcserrano@uloyola.es; Tel.: +34-955-641-600 (ext. 2579)

**Keywords:** biomass, polyols, catalysis, diols, selective deoxygenation, hydrogenolysis

## Abstract

The transition to a sustainable chemical industry necessitates efficient valorization of biomass, with polyols serving as versatile, renewable feedstocks. This comprehensive review, focusing on advancements within the last five years, critically analyzes the selective hydrogenolysis of key biomass-derived polyols—including glycerol, erythritol, xylitol, and sorbitol—into valuable diols. Emphasis is placed on the intricate catalytic strategies developed to control C–O bond cleavage, preventing undesired C–C scission and cyclization. The review highlights the design of bifunctional catalysts, often integrating noble metals (e.g., Pt, Ru, Ir) with oxophilic promoters (e.g., Re, W, Sn) on tailored supports (e.g., TiO_2_, Nb_2_O_5_, N-doped carbon), which have led to significant improvements in selectivity towards specific diols such as 1,2-propanediol (1,2-PD), 1,3-propanediol (1,3-PD), and ethylene glycol (EG). While substantial progress in mechanistic understanding and catalyst performance has been achieved, challenges persist regarding catalyst stability under harsh hydrothermal conditions, the economic viability of noble metal systems, and the processing of complex polyol mixtures from lignocellulosic hydrolysates. Future directions for this field underscore the imperative for more robust, cost-effective catalysts, advanced computational tools, and intensified process designs to facilitate industrial-scale production of bio-based diols.

## 1. Introduction

The increasing urgency to decarbonize the global economy has intensified efforts to transition from fossil-based resources to renewable alternatives. Among these, biomass stands out as the only renewable source of carbon capable of displacing petroleum in the production of fuels, chemicals, and materials [1,2,3]. Unlike fossil resources, biomass is globally distributed, annually replenishable, and compatible with closed-loop carbon cycles, thereby aligning with circular economy principles [4,5,6]. Moreover, biomass valorization reduces dependency on geopolitically volatile fossil reserves and offers pathways for regional economic development through agro-industrial integration [7]. Modern biorefineries try to mimic petrochemical refineries but aim to process lignocellulosic, starch-based, and residual biomass into high-value products through integrated fractionation and transformation platforms [8,9,10]. Among them, lignocellulosic biomass—composed primarily of cellulose, hemicellulose, and lignin—represents the largest and most sustainable feedstock pool, and it does not compete directly with food production [11,12,13].

One of the fundamental chemical differences between biomass and fossil carbon lies in the oxygen content. While fossil fuels are largely reduced hydrocarbons, biomass contains 30–50 wt% oxygen, typically in the form of hydroxyl, carboxyl, and carbonyl groups [14,15]. This high degree of oxygenation limits direct use of biomass intermediates as drop-in fuel and chemical replacements and requires careful deoxygenation strategies to tailor the functionality and stability [16,17]. In contrast to petrochemical routes that commonly involve controlled oxidative functionalization of hydrocarbons, biomass upgrading is primarily reductive via reactions such as hydrogenolysis, hydrodeoxygenation, and decarbonylation [18,19]. These transformations must be selective to avoid unwanted carbon loss, over-reduction, or unproductive by-products. Hence, designing catalytic systems that enable controlled C–O bond cleavage while avoiding C–C breaking reactions is crucial for achieving high atom economy and functional group specificity [20,21]. In this sense, selective deoxygenation routes are particularly relevant when targeting platform chemicals that aim to retain the carbon framework of the original biomass feedstock. These routes enable the controlled removal of excess oxygen functionalities—such as hydroxyl, carbonyl, or carboxyl groups—without inducing fragmentation or over-reduction, thereby preserving molecular complexity and maximizing carbon efficiency. This is especially critical for generating value-added chemicals that serve as intermediates in polymer, solvent, and fuel production. Unlike unselective deoxygenation, which often leads to CO_2_ release and loss of carbon atoms, selective strategies focus on modulating bond cleavage pathways through thermodynamic and kinetic control, tailored reaction conditions, and careful design of the catalytic environment. These approaches aim to enable de-functionalization while maintaining critical molecular features such as the chain length, branching, and stereochemistry, thereby facilitating downstream transformations into high-value compounds compatible with existing industrial processes.

**Figure 1:**
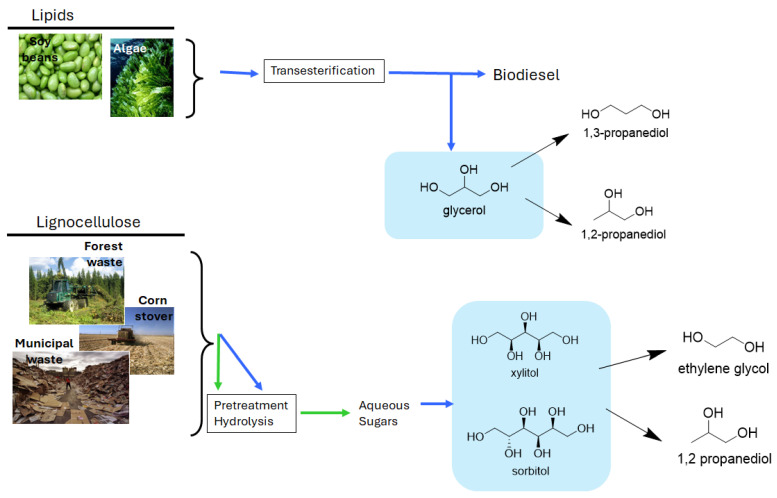
Schematic representation of biomass polyol production routes and their valorization to diols. Crude glycerol is obtained as a byproduct of biodiesel transesterification from triglyceride-rich feedstocks (e.g., soybean oil, algae). Lignocellulosic residues (e.g., forestry waste, agricultural stover, paper waste) undergo pretreatment and hydrolysis to yield C_5_–C_6_ polyols such as xylitol and sorbitol. These polyols are subsequently converted via selective hydrogenolysis into target diols (e.g., 1,2-PD, 1,3-PD and EG).

As shown in Figure 1, sugar alcohols, or polyols, constitute a prominent class of biomass-derived compounds that have garnered significant attention as chemical building blocks. These include glycerol, sorbitol, xylitol, erythritol, and mannitol, which are commonly produced mostly by catalytic hydrogenation, and to a lower extent microbial conversion, of the corresponding monosaccharides [22]. Glycerol is available in vast quantities as a by-product of biodiesel production, while sorbitol and xylitol are obtained from glucose and xylose, respectively, through well-established industrial routes. Importantly, polyols can be sourced not only from edible crops but also from second-generation biomass, including lignocellulosic hydrolysates derived from agricultural residues and woody materials [23]. These renewable resources contribute to the sustainability and economic feasibility of biorefinery concepts by integrating waste valorization with the production of value-added chemicals. Owing to their multifunctionality—typically containing three to six hydroxyl groups—polyols are ideally suited for catalytic transformations. Among the multiple products that can be obtained out of polyols, diols (e.g., ethylene glycol (EG), 1,2-propanediol (1,2-PD) and 1,3-propanediol (1,3-PD),) are integral to many sectors of the modern chemical industry. Their bifunctional nature enables their use as monomers, solvents, plasticizers, and intermediates in the synthesis of surfactants, resins, and pharmaceuticals [24]. Diols are widely produced at millions of tons annually to meet demand from the polyester and polyurethane markets. Currently, most industrial diols are derived from fossil-based routes, such as hydroformylation, acetylene chemistry, or the oxidation of alkenes. These routes are often energy-intensive and environmentally harmful, involving toxic intermediates and producing significant CO_2_ emissions [25]. In contrast, biomass-derived polyols offer an opportunity to access the same diol structures via more sustainable and potentially cost-competitive processes, reducing reliance on petroleum and minimizing process emissions. Moreover, biomass-derived diols can offer unique molecular architectures not readily accessible through petrochemical synthesis. These include branched, chiral, or cyclic diols with tailored properties for high-performance materials, such as bio-based polymers, specialty lubricants, and biodegradable surfactants [26].

In this review, a comprehensive overview of catalytic strategies for the selective deoxygenation of biomass-derived polyols into diols will be presented. Emphasis is placed on reaction mechanisms, selectivity challenges, and process integration considerations. Recent advances in heterogeneous systems and discussions on how catalyst design affects product distribution will be analyzed to explore the important implications of reaction media and process conditions. The objective is to provide a clear and critical perspective on the current state-of-the-art and to identify new unexplored approaches that can help pave the path toward scalable, sustainable diol production from renewable feedstocks.

## 2. Biomass-Derived Polyols: Characteristics and Reactivity

Polyols are hydrophilic, low-volatility compounds characterized by high boiling points and strong hydrogen-bonding capacity due to the presence of multiple hydroxyl groups. These properties confer excellent water solubility and hygroscopicity, making polyols well-suited for aqueous-phase catalytic processing [24]. Their relatively high viscosity, particularly for longer-chain polyols such as sorbitol and mannitol, can pose mass transfer limitations in heterogeneous systems and may require careful consideration in reactor design or solvent selection. Additionally, polyols have low vapor pressures and thermal stability under moderate processing conditions, allowing their transformation under hydrothermal or biphasic environments without significant volatilization losses. The extensive hydrogen bond network also affects crystallization behavior, influencing solid–liquid equilibrium in product recovery processes [26].

The industrial significance of biomass polyols is highlighted by their substantial global production volumes. In this sense, annual glycerol production consistently exceeds 4 million tons, while 2.4 million tons of sorbitol is produced annually, primarily via glucose hydrogenation. Similarly, xylitol, largely obtained from xylan-rich biomass, sees a substantial production rate of over 200,000 tons per year, driven by its applications as a sugar substitute. Beyond their theoretical potential, the practical relevance of bio-derived diols is increasingly evident in industrial applications. A notable example is BASF’s production of bio-based 1,4-butanediol, which is successfully utilized in the synthesis of polyesters, demonstrating a significant shift towards sustainable chemical manufacturing [24].

Biomass-derived polyols are highly reactive intermediates owing to their dense hydroxylation and flexible carbon skeletons. These molecules, including glycerol, sorbitol, and xylitol, typically contain three to six hydroxyl (–OH) groups, enabling a wide range of chemical transformations. The presence of multiple –OH groups enhances their nucleophilicity and allows them to engage in sequential or tandem reactions, such as hydrogenolysis, dehydration, isomerization, and oxidation. Their reactivity is influenced by the type (primary vs. secondary) and spatial orientation of the hydroxyl groups, which affect the bond strength, intermediate stability, and propensity for intramolecular rearrangements. As a result of their multifunctionality and high reactivity, biomass-derived polyols serve as versatile precursors to a diverse portfolio of chemical products via multiple catalytic and thermal pathways. Thus, under acidic or thermal conditions, polyols can undergo dehydration to yield unsaturated carbonyl compounds such as acrolein, hydroxyacetone, and levoglucosenone, which serve as intermediates in polymer and resin synthesis [27]. When processed in the presence of alcohols or dialkyl carbonates, transesterification reactions enable the formation of cyclic carbonates such as glycerol carbonate, a valuable monomer for non-isocyanate polyurethanes and green solvents. Oxidative pathways, often catalyzed by noble metals or enzymatic systems, convert polyols into carboxylic acids (e.g., glyceric acid, tartronic acid), ketoses, or aldoses, expanding their application in biodegradable polymers and fine chemicals [28]. Hydrogen transfer reactions, such as aqueous-phase reforming, also enable the cleavage of polyols into H_2_, CO_2_, and light alkanes—products that are central to energy applications but less suited for material synthesis due to carbon loss [29].

**Figure 2:**
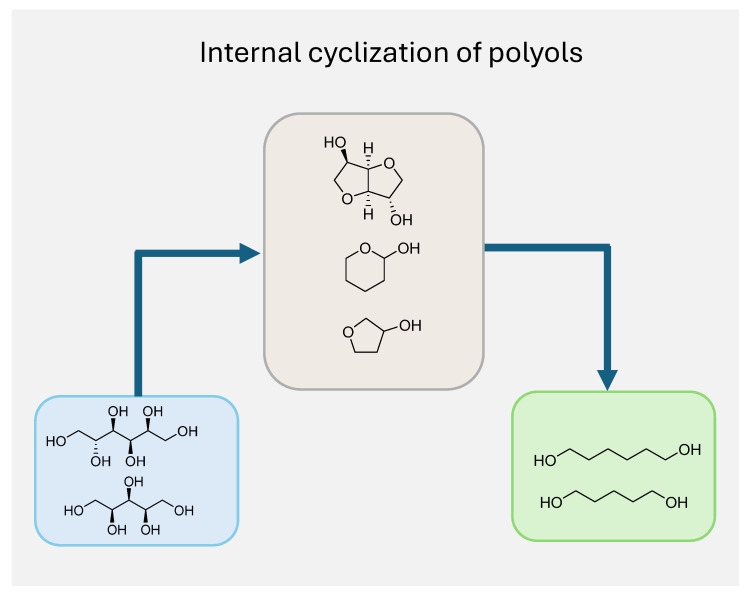
Internal cyclization pathways of polyols and their subsequent transformation. The figure illustrates how biomass-derived polyols (e.g., sorbitol, shown in the blue box) can undergo intramolecular cyclization via dehydration to form stable cyclic ethers (e.g., isosorbide and other cyclic ether derivatives, shown in the beige box). This cyclization represents a common side reaction that can reduce the yield of desired linear diols (green box).

Polyols are inherently prone to cyclization and etherification reactions, readily forming stable cyclic structures such as isosorbide, sorbitan, and polyether derivatives—key intermediates widely employed in the production of surfactants, plasticizers, and pharmaceuticals. These reactions are typically carried out via dehydration by acidic catalysts under controlled conditions, being highly favored by the proximity of hydroxyl groups for intramolecular ring closure [30]. The strong propensity to undergo internal cyclization via dehydration, particularly under acidic or elevated temperature conditions, represents the principal challenge in the selective deoxygenation of biomass-derived polyols to diols (Figure 2). This transformation competes directly with the desired hydrogenolysis of C–O bonds, diverting intermediates toward the formation of stable cyclic structures such as sorbitan and isosorbide. These cyclized products are thermodynamically favored and often more stable under reaction conditions, thereby significantly lowering the yield of linear diols. Moreover, once formed, these cyclic by-products are less prone to undergo deoxygenation, further complicating downstream processing. Addressing this barrier requires careful modulation of reaction conditions—such as the temperature, acidity, and residence time—and the use of catalysts that favor C–O bond cleavage while suppressing dehydration-induced ring closure. As will be detailed in the following sections, achieving this balance is critical for enhancing diol selectivity and maximizing carbon retention.

**Figure 3:**
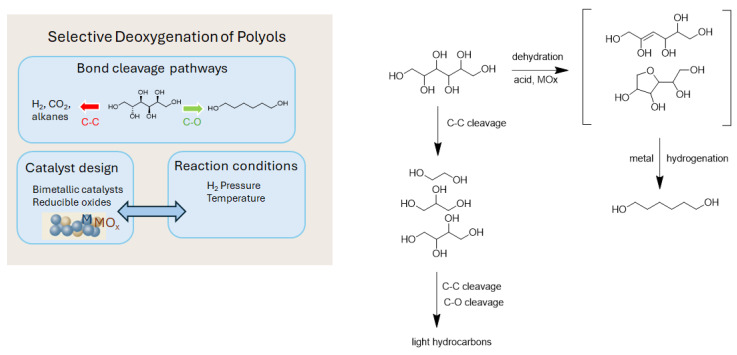
Conceptual overview of the main reaction pathways and selectivity challenges in the catalytic deoxygenation of biomass-derived polyols. The figure highlights competing C–O versus C–C bond cleavage routes, emphasizing the importance of catalyst design and reaction conditions in steering the transformation toward desired diol products.

The ability to control bond cleavage pathways (specifically between C–O and C–C bonds) is a defining factor in the design of efficient and selective catalytic deoxygenation processes of biomass polyols. While both bonds are targets for deoxygenation, the retention of the carbon backbone is crucial for maximizing molecular complexity and carbon efficiency. In this sense, selective cleavage of C–O bonds allows for targeted deoxygenation, leading to diols and other oxygen-reduced intermediates, whereas C–C bond scission results in carbon loss and product distribution skewed toward lower molecular weight compounds. Several factors influence the relative rates of C–O versus C–C bond cleavage in polyols, including the chemical structure of the polyol, the composition and structure of the catalyst, and the specific reaction conditions (Figure 3).

The chemical structure of the polyol plays a central role in determining whether C–O or C–C bond cleavage predominates during catalytic deoxygenation. Key features such as the number of carbon atoms, the distribution and type of hydroxyl groups, and the linearity or branching of the carbon backbone dictate both the thermodynamic stability of potential intermediates and the accessibility of reactive sites on the molecule. Thus, polyols with short, linear carbon chains (e.g., glycerol) generally favor selective C–O bond cleavage, particularly at terminal primary hydroxyl groups. In contrast, longer polyols such as sorbitol and xylitol possess more internal secondary hydroxyls, increasing the probability of C–O cleavage at those positions. Moreover, the location and combination of hydroxyl groups in polyols affect their reaction pathways. Polyols with more than six carbon atoms or with branching, such as those derived from sugar alcohol condensation reactions, present increased steric hindrance and reduced accessibility of hydroxyl groups, which hinders C–O activation and makes C–C bond cleavage more favorable [31]. Additionally, the presence of tertiary hydroxyl groups—less common in natural polyols—further suppresses C–O hydrogenolysis owing to the lack of β-hydrogen atoms necessary for effective bond scission, thereby steering reactivity toward dehydration or C–C cleavage [32].

The composition of the metallic phase in heterogeneous catalysts is critical in dictating selectivity toward C–O or C–C bond cleavage during polyol deoxygenation. Noble metals such as Pt, Ru, and Rh exhibit high hydrogenation activity but differ significantly in bond scission tendencies, depending on their electronic properties and coordination environment. For example, Pt is typically selective for C–O hydrogenolysis when paired with a second oxophilic metal such as Re or Mo, which introduces Lewis acidity and oxygen vacancy sites favorable for hydroxyl group activation [33]. Pt–Mo catalysts, in particular, show remarkable selectivity for 1,3-PD formation from glycerol by enabling dehydration at the secondary hydroxyl followed by hydrogenation at the terminal C=O intermediate [34]. In contrast, Ru tends to promote over-hydrogenolysis and C–C bond cleavage unless moderated by alloying with Sn, Re, or Co, which modulates the surface electronic structure and adsorption energies of key intermediates [35]. Ni-based systems, while economically attractive, require careful tuning through doping or bimetallic pairing to suppress excessive C–C fragmentation and promote diol formation [36]. Catalyst support materials also play a central role in tuning activity and selectivity by influencing metal dispersion, electronic properties, and acid–base interactions. Thus, reducible oxide supports such as TiO_2_, Nb_2_O_5_, and CeO_2_ introduce oxygen vacancies and redox-active surfaces that stabilize partially dehydrogenated intermediates and lower the barrier for C–O cleavage [37]. Rh–ReOx/TiO_2_, for instance, demonstrates enhanced selectivity toward 1,4-butanediol and 1,6-hexanediol from polyols by promoting selective activation of internal secondary hydroxyl groups while suppressing terminal dehydration or cyclization [38]. In contrast, acidic supports such as γ-Al_2_O_3_ and zeolites can promote undesired dehydration and cyclization, especially under elevated temperatures, reducing the selectivity to linear diols. Silica and carbon supports, although neutral, offer high surface areas and tunable hydrophobicity, which can modulate adsorption–desorption equilibria and favor sequential hydrogenolysis pathways with minimized side reactions. The combination of bifunctional sites—metallic for hydrogen activation and acidic or basic for C–O polarization—is crucial for steering the desired transformation and has become a core strategy in catalyst design. Beyond metal and support effects, structural parameters such as the particle size, morphology, and metal–support interface also influence bond scission outcomes. Nanoparticles below 5 nm tend to favor C–O cleavage due to higher densities of low-coordination sites, which stabilize alkoxide-like intermediates and reduce activation energies [39]. Additionally, strong metal–support interactions (SMSIs), particularly in systems such as Pt/TiO_2_ or Rh/CeO_2_, can modulate the charge transfer and local acidity at the interface, tuning adsorption modes and transition state energies. Core–shell and alloyed nanostructures have further enabled spatial separation of hydrogenation and activation sites, preventing over-hydrogenolysis and facilitating tandem reactions [40].

Apart from the composition of the metallic phase, the acid–base characteristics of the catalysts play a pivotal role in steering the reaction towards the desired products. For example, Brønsted acids facilitate dehydration steps (e.g., glycerol to acrolein), while Lewis acid sites, especially in oxophilic environments, enhance C–O bond polarization. Simultaneously, basic sites are essential for retro-aldol cleavage and stabilization of carbonyl intermediates, particularly in the transformation of higher polyols such as sorbitol and xylitol. The nature of metal–support interactions, including SMSI effects in systems such as Pt/TiO_2_ or Ni/CeO_2_, directly influences electron density at the metal centers, modifies adsorption energies, and alters reaction pathways [40]. Additionally, alloying strategies—e.g., Pt–Re, Ni–Cu, Pd–Zn—allow fine-tuning of d-band centers of the metals and intermediate binding strengths, mitigating over-hydrogenolysis and favoring selective C–O cleavage [33]. More recently, atomically dispersed metals and sub-nanometric clusters have introduced site-specific reactivity profiles; single-atom catalysts (SACs), in particular, offer unsaturated coordination sites and high metal utilization, enabling highly selective activation of hydroxyl groups under mild conditions. These properties render SACs and alloyed systems attractive alternatives to conventional bulk metal catalysts for steering selectivity in complex reaction networks.

The reaction temperature is one of the most critical parameters controlling the balance between C–O and C–C bond cleavage during polyol deoxygenation. At moderate temperatures (393–453 K), catalytic systems such as Pt–Mo or Cu–Re selectively cleave C–O bonds while preserving the carbon skeleton, favoring the formation of diols such as 1,3-PD and 1,2-butanediol from glycerol or xylitol [33]. As the temperature increases beyond 473 K, thermal energy facilitates C–C scission pathways such as retro-aldol condensation or β-scission, leading to fragmentation into smaller alcohols and gases. Thus, the temperature sensitivity of different catalytic systems must be considered to avoid undesired over-conversion or carbon loss and to tailor activation energy barriers toward C–O selectivity [36].

The hydrogen partial pressure also significantly affects selectivity by modulating the surface concentration of hydride species and influencing intermediate stability. Low hydrogen pressures (e.g., <10 bar) may be insufficient for efficient hydrogenation of C–O cleaved intermediates, leading to accumulation of dehydrated or unsaturated species that undergo further side reactions such as cyclization or cracking. Conversely, excessively high hydrogen pressures (>50 bar) can over-saturate metal surfaces, promoting over-hydrogenation and C–C bond cleavage, especially in systems with high metal loading or small particle sizes. Recent studies on Ru–Re/TiO_2_ catalysts have shown that an optimal H_2_ pressure window (20–30 bar) maximizes diol yields by balancing the rates of hydrogenation and dehydration suppression [41]. Additionally, hydrogen availability influences the equilibrium of reversible hydrogenolysis reactions and can determine the dominant cleavage pathway, depending on the type of hydroxyl group and catalyst interface.

The solvent environment and phase are also crucial for steering selectivity. Aqueous-phase systems are often preferred due to the hydrophilic nature of polyols, but water can promote acid-catalyzed dehydration and cyclization, especially under high-temperature conditions, leading to formation of cyclic ethers such as isosorbide or sorbitan. In contrast, organic solvents such as alcohols or glymes reduce water activity and can suppress undesired intramolecular rearrangements. Solvent polarity also modulates intermediate solvation and transition state stabilization, directly influencing the preference for C–O over C–C cleavage. Furthermore, biphasic systems (e.g., water–methyl isobutyl ketone) allow in situ extraction of diol products, minimizing secondary reactions and improving selectivity [42].

In the sections that follow, the review focuses primarily on the selective deoxygenation of three representative polyols: glycerol (C_3_), xylitol (C_5_), and sorbitol (C_6_). These compounds were selected due to their distinct chain lengths, widespread industrial availability, and prominence in the recent literature related to hydrogenolysis and C–O bond activation strategies. This selection allows a detailed comparative analysis across molecular sizes and hydroxyl group configurations, highlighting how structure–reactivity relationships and catalytic strategies evolve with polyol complexity. Other polyols such as erythritol and mannitol are discussed in brief within Section 4, particularly in relation to current research gaps and opportunities for further development.

## 3. Catalytic Deoxygenation of Polyols to Diols

### 3.1. Glycerol

The selective hydrogenolysis of glycerol into propanediols, namely 1,2-PD and 1,3-PD, has emerged as a strategic route within biomass valorization schemes, enabling the production of high-value C_3_ diols from renewable feedstocks. With regards to 1,3-PD, initial dehydration of glycerol typically takes place at the secondary hydroxyl group, resulting in the formation of 3-hydroxypropanal (3-HPA) or acrolein intermediates, depending of the catalysts used and the reaction conditions [43,44,45]. Acrolein is a highly reactive, α,β-unsaturated aldehyde that is particularly promoted over bifunctional (metal–acid) catalytic systems, such as those based on Pt–WO_x_ or Ir–ReO_x_, where acid sites facilitate the dehydration of glycerol to acrolein followed by rapid hydrogenation over adjacent metallic sites (Pt or Ir) to yield 1,3-PD. The effectiveness of this tandem pathway relies on a delicate balance between the number and strength of the acidic sites (i.e., they must be strong enough to promote the dehydration to acrolein), while the hydrogenation sites must be sufficiently active to prevent the polymerization or coking of acrolein, thereby ensuring its swift conversion to the desired diol. This strategy offers a C–O bond cleavage mechanism that circumvents excessive C–C scission, leading to improved carbon retention and higher selectivity towards 1,3-PD (Table 1).

**Figure 4:**
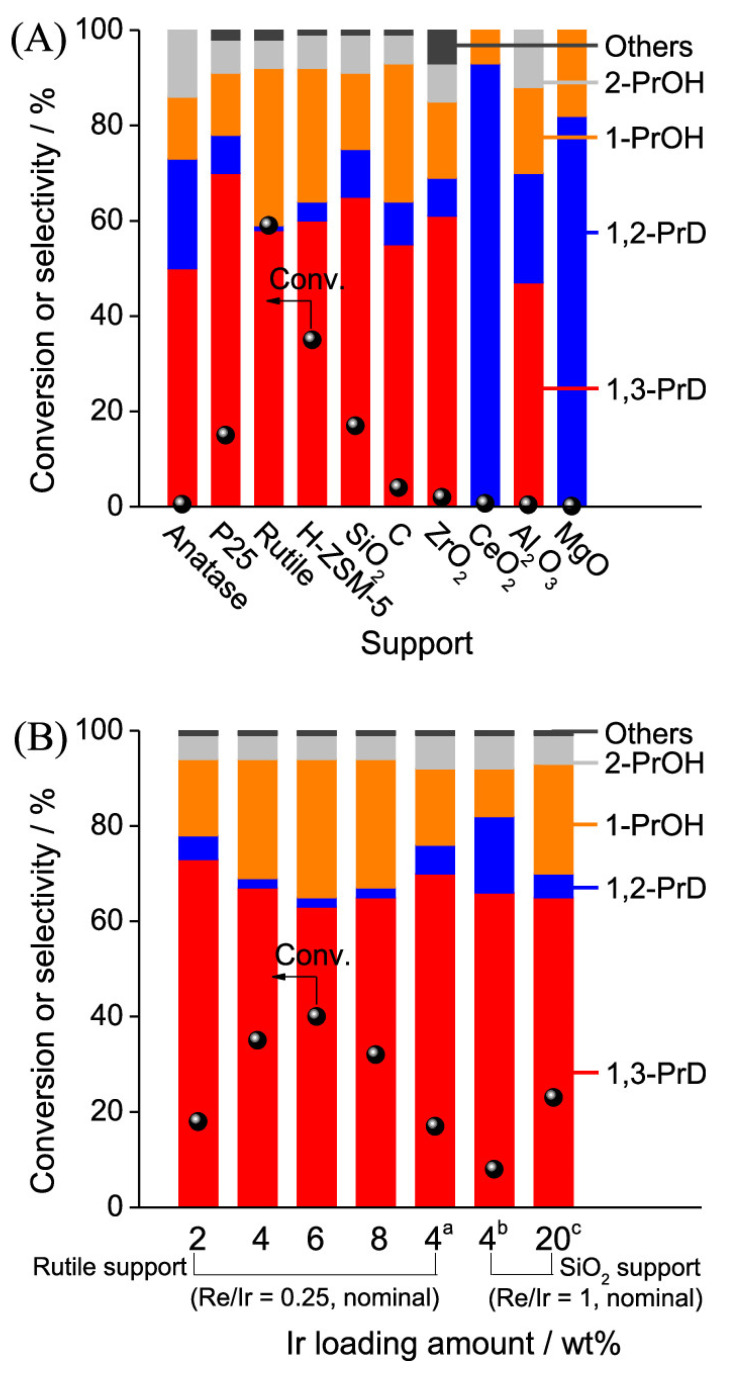
Performance of Ir-ReOx catalysts in glycerol hydrogenolysis: (**A**) impacts of different support materials; (**B**) effects of varying Ir loading rates. Both studies were conducted under specific reaction conditions (8 MPa, 393 K, 8 h), with variations in reaction time and reduction conditions noted for certain experiments [43]. ^a^ Ir(NO_3_)_4_ as Ir precursor. ^b^ Ir-ReO_x_/SiO_2_ (4 wt %Ir, Re/Ir = 1, nominal), reduced in water at 473 K; ^c^ Ir-ReO_x_/SiO_2_ (20 wt % Ir, Re/Ir = 1, nominal), reduced in water at 473 K.

Among the most effective systems reported, Re- and W-promoted catalysts supported on metal oxides such as TiO_2_ and Al_2_O_3_ have demonstrated exceptional promise. Liu et al. developed a ReO_x_–Ir/TiO_2_ catalyst that afforded a glycerol conversion of 80% and a 1,3-PD selectivity rate near 50% under 393 K and 8 MPa H_2_ [43]. Mechanistic studies of these Ir−ReO_x_ catalysts suggest a direct hydrogenolysis pathway where Re–OH sites promote absorption of the terminal hydroxyl group of glycerol, while active hydride species, generated on neighboring Ir atoms, attack the C_2_ position of the adsorbed glycerol, leading to selective C–O bond cleavage and 1,3-PD formation. In this study, the Ir−ReO_x_ interface is proposed as the active site, with the interaction between Ir metal particles and partially oxidized ReO_x_ clusters being crucial to achieve high activity and 1-3-PD selectivity. Interestingly, Ir-ReO_x_ supported in rutile TiO_2_ demonstrated the highest activity and selectivity to 1,3-PD among various supports, including commonly used SiO_2_ (Figure 4A), whereas anatase showed almost no activity. H-ZSM-5 also showed good activity, potentially due to densely located Ir particles as a result of its small external surface area and proper support acidity. Other supports such as carbon, ZrO_2_, CeO_2_, Al_2_O_3_, and MgO were less effective. Most catalysts, except those on basic supports (e.g., CeO_2_, MgO), maintained high selectivity to 1,3-PD. A volcano-type dependency of glycerol conversion with the Ir loading (2–8 wt%) was observed for Ir-ReO_x_/rutile, peaking at 6 wt% Ir (Figure 4B). This superior performance of Ir-ReO_x_/rutile was attributed to its intrinsic properties rather than solely its surface area. The Ir precursor was also found to have a large influence on the hydrogenolysis activity, with H_2_IrCl_6_ leading to higher activities.

Pt–WO_x_/SBA-15 catalysts have been shown to reach 1,3-PD selectivities as high as 45%, with Priya et al. attributing this performance to enhanced acid–metal synergism resulting from tungsten incorporation, even at trace levels. The enhanced 1,3-PD selectivity over Pt–WO_x_/SBA-15 catalysts was attributed to the presence of Brønsted acid sites, introduced by the WO_x_ species, which were essential for the dehydration of the glycerol secondary hydroxyl group to 3-HPA. Subsequent hydrogenation of 3-HPA occurs over the Pt metallic sites, completing the two-step dehydration–hydrogenation mechanism that favors 1,3-PD formation [44].

Guadix-Montero et al. further demonstrated that supported Ru nanoparticles with an average size of ca. 2 nm exhibit moderate selectivity toward 1,3 PD, reaching values around 10–13% under aqueous-phase hydrogenolysis conditions (438 K, 20 bar H_2_), thereby underscoring the role of particle size and metal–support interactions in tuning the product distribution [45]. Monometallic Ru catalysts were found to favor C–C bond cleavage, leading to a number of C_1_ and C_2_ products. This preference for C–C scission stems from the Ru intrinsic electronic and geometric properties that favor the activation and fragmentation of the glycerol backbone. When combined with Pt or Pd in bimetallic nanoparticles (e.g., RuPd or RuPt), significant enhancement of the selectivity towards C–O hydrogenolysis was found. Ru alloys lead the reaction towards C_3_ diols, reaching selectivity rates as high as 58 and 65% for RuPd and RuPt catalysts, respectively, versus 30% observed with monometallic Ru. Density functional theory (DFT) studies revealed RuPd to promote the thermodynamically favorable dehydration reaction involving an acetol intermediate, which subsequently hydrogenates to 1,2-PD. On the other hand, RuPt alloys were found to promote formation of 1,3-PD selectively. This study is interesting in that it demonstrates that the specific alloying elements and their resultant electronic and structural modifications enable a fine-tuning of the active sites, allowing for the selective activation of desired C–O bonds. Wang et al. demonstrated that selecting appropriate solvent environments strongly influences product distribution in glycerol hydrogenolysis over Cu–ZnO catalysts. Transitioning from pure water to alcohol co-solvents (such as methanol or ethanol) significantly suppressed cyclic and ether byproducts and increased 1,2-PD selectivity from ~65% to ~80%, illustrating the critical role of solvent effects in optimizing linear diol yield [46].

Efforts to reduce reliance on noble metals have led to the development of Cu- and Ni-based bimetallic catalysts alloyed with oxophilic metals (Re, Mo, W), which show improved activity and selectivity in glycerol hydrogenolysis [25]. In a parallel study, Zhang et al. investigated glycerol hydrodeoxygenation over NiMo_2_C/Al_2_O_3_ catalysts and reported ca. 61% selectivity to 1,2 PD with high carbon balance under mild hydrogenolysis conditions [47]. The improved catalytic performance of the NiMo_2_C/Al_2_O_3_ catalysts was ascribed to a synergistic interaction between the deoxygenation activity of NiMo_2_C sites and the acid–base properties of the Al_2_O_3_ support. This synergy facilitates efficient C–O bond cleavage and selective hydrogenation of intermediate species, thereby preserving the carbon backbone and leading to higher 1,2-PD selectivity rates. The improved catalytic performance was ascribed to the synergistic interaction between the deoxygenation activity of NiMo_2_C and the acid–base properties of the Al_2_O_3_ support. Thus, Al_2_O_3_ favored dehydration of glycerol to acetol (hydroxyacetone) and 3-HPA. Acetol was a direct precursor to 1,2-PD upon hydrogenation. The role of the NiMo_2_C phase was found to be critical in the subsequent hydrogenation of these highly reactive dehydration products. The distinct acidity profiles of the catalysts, with NiMo_2_C/Al_2_O_3_ possessing a higher fraction of medium and strong acid sites compared to pure Al_2_O_3_, further support this bifunctional mechanism.

García-Fernández et al. showed that Pt/WO_x_/Al_2_O_3_ catalysts with adjusted tungsten oxide loading—and by extension, TiO_2_ analogues—stabilize metal–acid interfaces and minimize side reactions [48]. Their study reports selectivity to 1,3-PD of 38.5%, attributing enhancements to controlled Brønsted acidity and interfacial stabilization. The enhanced selectivity to 1,3-PD was explained based on a precisely controlled bifunctional mechanism involving both metallic platinum and tungsten oxide species on the alumina support. The tungsten oxide, particularly in its polytungstate form, was found to provide crucial Brønsted acidity essential for the initial dehydration of glycerol to 3-HPA. The close proximity and stabilization of the metal–acid interfaces, achieved by optimizing the WO_x_ loading, were found to be critical for minimizing undesired side reactions and ensuring the swift conversion of 3-HPA to 1,3-PD.

As indicated in the previous section, reaction parameters, particularly hydrogen pressure and temperature, exert substantial influence on product distribution. Thus, high hydrogen pressures (>5 MPa) typically favor 1,2-PD due to its kinetically preferred formation via hydroxyacetone intermediates, as shown by Liu et al. [49]. Conversely, 1,3-PD formation benefits from moderate hydrogenation conditions and higher acidity, which promote acrolein intermediates [50]. Mechanistic studies suggested that the presence of both Lewis acid and hydrogenation sites is essential to direct C–O bond cleavage over C–C scission, thereby preserving the carbon chain and enhancing 1,3-PD yields [51]. Strategies incorporating biphasic media co-solvents have also been shown to shift selectivity profiles by reducing over-hydrogenation and C–C cleavage reactions during the hydrogenolysis of glycerol. Thus, the use of high-boiling polar co-solvents (e.g., sulfolane) was found to suppress undesired pathways and favored 1,3-PD formation, highlighting how the solvent choice can strategically direct the product distribution [52].

With regards to the nature of the active sites, operando and theoretical investigations revealed that acid–metal domains generated under hydrogen-rich conditions are crucial to direct hydrogenolysis to 1,3 PD via acrolein formation and subsequent activation [53]. Similar findings have been reported with Ir-ReO_x_ [43] and Pt/WO_x_ catalysts (~46% 1,3-PD) [54]. The stability and recyclability of the catalysts during glycerol hydrogenolysis have shown promising results, considering the harsh hydrothermal conditions at which these reactions take place and potential metal leaching issues. Thus, Pt–WO_x_/Al_2_O_3_ catalysts preserved their structural integrity and maintained high 1,3-PD selectivity after multiple regeneration cycles, with minimal metal leaching in aqueous-phase conditions [53]. Sulfated Ru/ZrO_2_ catalysts retained ~48% 1,3-PD selectivity across five batch recycles, demonstrating high recyclability and suppressed carbon loss [50]. Coke formation was minimized over egg shell Ir/ReO_x_ catalysts, thereby enhancing the catalyst’s longevity under operational harsh conditions [55]. PtCu single-atom alloys (SAAs) showed noticeable stability after five consecutive glycerol hydrogenolysis cycles, the catalyst maintained >99% conversion and >98% 1,2-PD selectivity, with only minor performance declines attributable to superficial CuO formation, fully recovered via hydrogen reduction [56].

**Figure 5:**
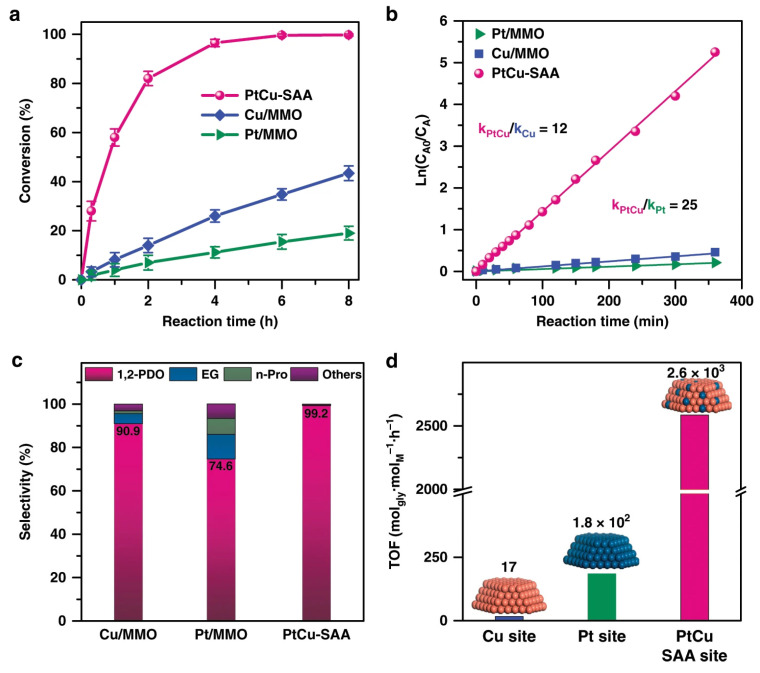
Performances of PtCu-SAA, Pt, and Cu nanoparticles supported on mixed metal oxides (MMOs) in the hydrogenolysis of glycerol to 1,2-PD: (**a**) glycerol conversion with reaction time; (**b**) a kinetic analysis using a first-order plot; (**c**) product distribution to 1,2 PD, EG, n-propanol and others; (**d**) TOF calculated based on initial glycerol conversion and exposed active sites. Reaction conditions: 10 wt% glycerol solution, 0.14 g of catalyst, 473 K, and 2.0 MPa H_2_ pressure, with data averaged from three runs [56].

PtCu–SAA catalysts significantly outperformed monometallic Pt/MMO and Cu/MMO in glycerol hydrogenolysis to 1,2-PD (Figure 5a). The PtCu–SAA catalyst showed a reaction rate several orders of magnitude higher than the Cu/MMO or Pt/MMO monometallic catalysts (Figure 5b). This higher catalytic activity was attributed to a cooperative, synergistic effect between the Cu and Pt components. PtCu–SAA achieves exceptional conversion (99.6%) and selectivity (99.2%), even at low temperatures (Figure 5c). The PtCu–SAA catalyst showed extraordinarily high TOF values, far surpassing both monometallic Cu/MMO and Pt/MMO catalysts (Figure 5d). The achieved TOF for PtCu–SAA was among the highest documented to date in the literature. PtCu–SAA also demonstrated good reusability over five cycles, with only minor deactivation attributed to slight CuO formation. Subsequent reduction effectively restores its high catalytic activity and recyclability for further cycles. Given the uncertain stability of single-metal atoms and related materials under harsh temperature and hydrothermal conditions, this represents a significant achievement. These results have stimulated further exploration of SAA systems for the conversion of biomass feedstocks into chemicals, including polyols selective hydrogenolysis [57,58]. Thus, the unique electronic and geometric structures of SAAs provide opportunities to optimize the cleavage of C–O bonds and minimize undesirable C–C cleavage side reactions in complex biomass-derived molecules such as glycerol, paving the way for more efficient and sustainable chemical production. These studies have highlighted the pivotal role of acrolein as a key intermediate in the hydrogenolysis of glycerol to 1,3-PD. For instance, in certain bifunctional catalytic systems, particularly those based on Pt–WO_x_ or Ir–ReO_x_, glycerol undergoes selective dehydration to acrolein over acid sites at the secondary hydroxyl position, followed by hydrogenation of the dehydrated intermediate to 1,3-PD over metal sites. This tandem pathway offers a C–O bond cleavage mechanism that avoids excessive C–C scission and improves carbon retention. The stabilization of acrolein intermediates is facilitated by the presence of acidic sites capable of promoting dehydration, while adjacent hydrogenation sites prevent polymerization or coke formation by providing a fast saturation under high hydrogen pressure. The ability to steer glycerol transformation through the acrolein route is particularly sensitive to the balance of Lewis acidity and hydrogenation capacity, reinforcing the importance of precise tuning of catalyst bifunctionality.

Numerous studies have emphasized the efficacy of Pt–WO_x_-based catalysts for the selective hydrogenolysis of glycerol to 1,3-PD, with particular focus on the critical interplay between tungsten oxide domains and platinum nanoparticles (Table 1). The use of Pt/WO_x_ catalysts, often supported on TiO_2_ or its polymorphs, has consistently resulted in high 1,3-PD selectivities under aqueous-phase reaction conditions. For instance, Zhao et al. [59] reported a 1,3-PD selectivity rate of 51% using a Pt/WO_x_ catalyst under 433 K and 5.0 MPa H_2_ of pressure, attributing the activity to the presence of positively charged Pt sites and strong metal–acid bifunctionality. The positively charged Pt species were particularly effective in adsorbing hydrogen and facilitating its heterolytic dissociation, which significantly enhanced their hydrogenation activity. This, coupled with an optimized interface between Pt and WO_x_ species, led to the efficient hydrogenation of intermediate 3-HPA to 1,3-PD.

Similarly, Zeng and co-workers [60] demonstrated the performance of Pt–WO_x_ supported on rutile TiO_2_, achieving a selectivity rate of 51.2% at 150 °C and 4.0 MPa H_2_, highlighting the structural advantages imparted by the rutile phase over anatase or amorphous supports. The superior catalytic performance of the Pt–WO_x_ catalyst supported on rutile TiO_2_ was attributed to the rutile’s ability to facilitate faster hydrogen spillover from the Pt nanoparticles to the reaction intermediate on the WO_x_ species. This efficient hydrogen spillover was found to be crucial for achieving rapid hydrogenation to 1,3-PD.

**Table 1:** Summary of recent catalytic systems for the selective hydrogenolysis of glycerol to 1,2- and 1,3-PD.

Catalyst System	Reaction Conditions	Selectivity to PDs	Reference
Ir–ReO_x_/TiO_2_	160 °C, 3 MPa H_2_, aqueous, batch reactor, 4 g (43 mmol) of glycerol in 2 g of water, 150 mg of 4 wt% Ir Ir-ReO_x_/TiO_2_; 4 h reaction time; TOF 830 h^−1^	up to 70% (1,3-PD)	[43]
Pt–WO_x_/SBA-15	210 °C, 0.1 MPa H_2_, aqueous, fixed bed reactor; 10wt% glycerol in water; 0.5 g of catalyst; WHSV: 1.02 h^−1^	45% (1,3-PD)	[44]
Ru NPs	160 °C, 2 MPa H_2_, aqueous; batch reactor; 5 wt% of glycerol in water, metal-to-substrate molar ratio of 1:200; 16 h reaction time;	15% (1,3-PD)	[45]
Ni–Mo_2_C/Al_2_O_3_	200 °C, 3 MPa H_2_, aqueous; batch reactor; 95 wt% of glycerol in water, 1.2 g of catalyst; 6 h reaction time;	61% (1,2 PD)	[47]
Pt–WO_x_/Al_2_O_3_	240 °C, 5 MPa H_2_ aqueous; batch reactor; 5 wt% of glycerol in water, 0.3 g of catalyst/g glycerol; 4 h reaction time;	40% (1,3-PD),	[53]
Pt/WO_x_	140 °C, 1 MPa H_2_ aqueous; fixed bed reactor; 5 wt% of glycerol in water; 75 h reaction time; LHSV: 1 h^−1^	46% (1,3-PD), 7% (1,2-PD)	[54]
egg-shell Ir/ReO_x_	130 °C, 8 MPa H_2_, aqueous; fixed bed reactor; 5 wt% of glycerol in water, 1.5 g of catalyst; 36 h reaction time	31% (1,3-PD)	[55]
Pt–WO_x_/r–TiO_2_	150 °C; 4 MPa H_2_, batch reactor; 30 wt% of glycerol in water, 0.2 g of catalyst; 24 h reaction time	51.2% (1,3-PD)	[60]
Pt/WO_x_	160 °C, 5.0 MPa H_2_, batch reactor; 30 wt% aqueous glycerol; 0.2 g of catalyst; 24 h reaction time	51% (1,3-PD)	[59]

### 3.2. Xylitol

Xylitol, a C_5_ polyol derived from hemicellulose via the hydrogenation of xylose, can serve as a promising biomass platform molecule for the production of short-chain diols such as EG and 1,2-PD. As in the case of glycerol, fine control of C–C and C–O bond cleavage reactions is crucial to minimize over-hydrogenation and the formation of undesired side products such as lactic acid or C_1_–C_4_ fragments [61]. The mechanism of xylitol hydrogenolysis generally takes place via a cascade sequence, with a first dehydrogenation of the terminal alcohol to generate reactive carbonyl intermediates followed by retro-aldol cleavage. Retro-aldol cleavage typically follows an initial dehydrogenation of the terminal alcohol group of xylitol to form reactive carbonyl intermediates. These carbonyl intermediates then undergo retro-aldol fragmentation, often catalyzed by basic sites, yielding smaller aldehyde or ketone fragments (e.g., glycolaldehyde, glyceraldehyde). These fragments are subsequently hydrogenated over metallic sites to produce the desired C_2_ and C_3_ glycols. The delicate balance and synergy between basic sites (for retro-aldol cleavage) and metallic sites (for hydrogenation) are crucial for controlling selectivity and preventing excessive C–O or further C–C scission.

Ruthenium-based catalysts are particularly active for the initial dehydrogenation and terminal hydrogenation steps, while the cleavage of C–C bonds is promoted by the presence of basic co-catalysts such as MnO or CaO. The synergy between the metal and base sites is key to enable the selective formation of polyol products. Ca(OH)_2_ is commonly used in homogenous basic catalysts, given the Mn leaching issues in the aqueous phase [62]. The presence of a basic component is key, since unpromoted Ru/C favored side over-hydrogenation to C_5_ alditols (e.g., arabitol, threitol) and light alkanes, thereby limiting the yield of desired glycols. Thus, the addition of a basic promoter such as Ca(OH)_2_ promotes cleavage of the C_5_ polyol into smaller fragments such as glycolaldehyde, which are then readily hydrogenated by the metallic ruthenium sites to produce C_2_ and C_3_ glycols (e.g., EG and propylene glycol). This synergistic action between the metal (for hydrogenation) and the base (for retro-aldol fragmentation) outcompetes the undesired side reactions, significantly enhancing the yield of target glycols. Reaching an optimal concentration of the basic promoter is critical, as demonstrated by the direct correlation between Ca(OH)_2_ loading and glycol selectivity.

Non-noble catalysts such as Ni/CeO_2_-TiO_2_ have also demonstrated promising selectivity values, reaching glycol selectivity rates as high as 80%, with lactic acid formation being suppressed under aqueous conditions [63]. Here, CeO_2_ enhanced the stability of nickel by resisting leaching and sintering. This was evidenced by the significantly smaller crystallite size of Ni (approximately 8.5 nm) in the presence of cerium, compared to their larger counterparts without cerium (19.4 nm). This reduction in particle size was attributed to a stronger interaction between the Ni nanoparticles and the meso-Ce–TiO_2_ support, which promotes better dispersion of Ni and inhibits its aggregation during calcination and reaction, as supported by XRD and H_2_-TPR analyses. Furthermore, the stability against leaching was demonstrated by the sustained catalytic performance over multiple reaction cycles, where the total yield of diols decreased only slightly (to 78% after 8 runs). In contrast, catalysts prepared by conventional impregnation methods, lacking this intimate Ce-Ni interaction, showed severe deactivation as a result of the leaching of Ni species. Further explanations of the effect of the Ce–Ti–O framework on the substrate interaction or the kinetics of the retro-aldol condensation pathways are lacking.

Bimetallic systems such as Pd–Pt/TiO_2_ were used under catalytic transfer hydrogenolysis (CTH) conditions. Thus, using formic acid as an in-situ hydrogen source under inert N_2_, the bimetallic catalyst achieved near-complete xylitol conversion with over 40% selectivity to C_2_–C_3_ glycols [64]. Importantly, the synergistic behavior between Pd and Pt was found to be size-sensitive, with optimal selectivity being achieved by bimetallic particles of ca. 4 nm in diameter. This size-dependent synergy was found to be critical for balancing the rates of H_2_ generation (via C–H cleavage of the hydrogen donor) and the subsequent C–C/C–O cleavage of xylitol intermediates, which includes the retro-aldol pathway. This work is interesting in that it paves the way for carrying out hydrogen-free hydrogenolysis using water soluble formic acid or even hydrogen donors such as butanol [65]. Li [65] and Liu [66] and independently demonstrated the efficacy of bimetallic Ni–Cu-based catalysts for the selective hydrogenolysis of xylitol to short-chain glycols under base-free conditions. In particular, Liu et al. [66] reported that NiCu/SiO_2_ catalysts with well-alloyed metal phases achieve over 85% xylitol conversion and 80% combined selectivity to EG and 1,2-PD at 493 K and 8 MPa H_2_, highlighting a synergistic effect where Ni facilitates C–C cleavage and Cu suppresses over-hydrogenation. The overall process involved three key mechanistic steps under basic conditions: (1) an initial, kinetically relevant dehydrogenation of xylitol to xylose over the metallic sites; (2) subsequent C–C bond cleavage of xylose via retro-aldol condensation catalyzed by a base promoter (e.g., Ca(OH)_2_); (3) hydrogenation of the resulting carbonyl intermediates (glycolaldehyde, glyceraldehyde) to the target glycols on the metal surfaces. In this bimetallic catalyst, Cu is efficient in C–OH hydro-dehydrogenation (i.e., the first step of xylitol dehydrogenation) and selectively cleaves C–O bonds, while Ni possesses high hydrogen activation capability and facilitates C–C bond scission. Thus, decoration of Cu nanoparticles with Ni resulted in the formation of Ni surface-enriched Cu–Ni alloy sites, as evidenced by X-ray diffraction (XRD), high-resolution transmission electron microscopy (HRTEM), and X-ray photoelectron spectroscopy (XPS) results. These alloy sites enhanced the reducibility of the bimetallic catalyst, promoting the formation of active metal sites with a lower barrier for H_2_ activation. The electronic effects within the Cu–Ni alloy, involving electron transfer from Ni to Cu, allowed further tuning of the bonding properties of the alloy surfaces, leading to enhanced catalytic activity. The high hydrogenation activity of the alloy was also found to improve the stability and reusability of the NiCu–SiO_2_ system by minimizing the accumulation of coke precursors.

**Figure 6:**
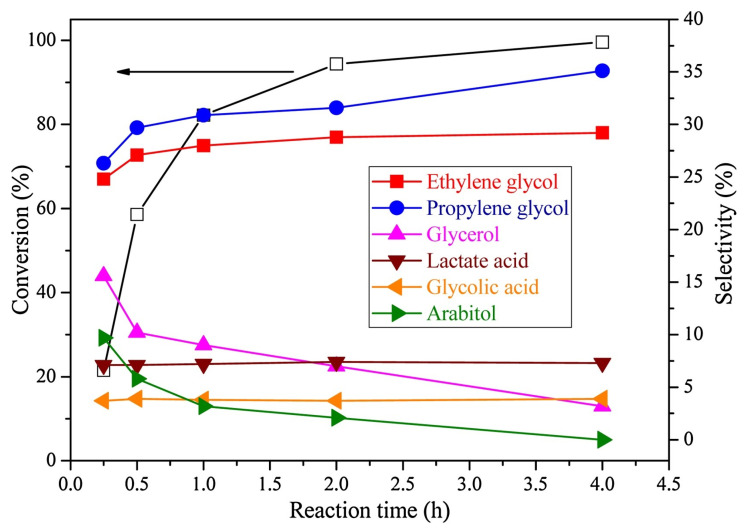
Conversion of xylitol and selectivity of products during xylitol hydrogenolysis over a 10Ni80Cu–SiO_2_ catalyst. The reaction was conducted with a 10 wt% xylitol solution, 0.144 g of active metal, 0.600 g of Ca(OH)_2_, at 473 K and 6 MPa H_2_ [66].

The optimization of xylitol hydrogenolysis parameters was performed to maximize ethylene and propylene glycol yields [66]. Increasing the xylitol concentration was found to decrease both conversion and glycol selectivity rates, indicating that a higher metal catalyst-to-substrate ratio is beneficial. Both xylitol conversion and glycol selectivity rates improved with increasing H_2_ pressure (4–8 MPa). The optimal reaction temperature was 473 K, and higher temperatures (493 K) caused catalyst deactivation. As shown in Figure 6, xylitol conversion rapidly increased in the first hour, reaching near completion after 4 h. Similarly, ethylene and propylene glycol selectivity rates quickly rose before levelling off. The glycerol and arabitol selectivity rates decreased over time, confirming their roles as intermediates. These findings highlight that high glycol yields can be achieved with the optimal catalyst-to-reactant ratios, temperature, H_2_ pressure, and reaction time. The optimum 10Ni80Cu–SiO_2_ catalyst achieved an 81.0% combined yield of ethylene and propylene glycol with full xylitol conversion under optimized conditions, surpassing many noble metal catalysts reported previously. Li et al. [65] used bimetallic Cu–Ni–ZrO_2_ catalysts to carry out the hydrogenolysis of xylitol, achieving 81% yield of glycols in aqueous media without adding solid bases. The ZrO_2_ support provided acid–base bifunctionality to promote retro-aldol fragmentation.

Solvents can also be used to modulate reaction kinetics and product selectivity. Thus, aqueous–organic mixtures, particularly H_2_O/MeOH (90:10), have been shown to increase glycol yields and suppress epimerization or over-reduction pathways, due to modified adsorption behavior and competitive inhibition of alkoxide formation. However, excessive alcohol contents (>20%) cause reduced activity and can lead to deactivation via coke deposition on metal sites, especially for Ru catalysts. MnO addition mitigates these effects by suppressing alcohol dehydrogenation and blocking sites responsible for coke precursors [67]. The addition of MnO was found to mitigate undesirable effects by suppressing alcohol dehydrogenation and blocking sites generating coke precursors. Thus, MnO ensured that the primary retro-aldol cleavage and subsequent hydrogenation steps are not hindered by catalyst deactivation. As in the case of glycerol, the catalyst stability in xylitol hydrogenolysis is challenged primarily by metal leaching, coking, and particle sintering. Ru/MnO/C underwent significant Mn leaching (up to 70%) in aqueous media, which correlates with reduced glycol selectivity and minor lactic acid formation over time [62]. Ni-based catalysts supported on CaO or CeO_2_ showed better structural stability and lower leaching rates, maintaining their performance over multiple cycles [31,63]. Table 2 summarizes the above-described results on xylitol hydrogenolysis.

### 3.3. Sorbitol

Sorbitol, a six-carbon sugar alcohol readily obtained via the hydrogenation of glucose, has a chemical structure that makes it an ideal platform molecule for chemical conversion into high-value products such as EG, 1,2 PD, 1,4-sorbitan, and other lower polyols including glycerol. However, the high reactivity of sorbitol makes it challenging to control the selectivity to targeted products, which typically results in modest selectivity rates (Table 3). Retro-aldol condensation is a critically important pathway during the hydrodeoxygenation of sorbitol, facilitating C–C cleavage to ethylene and propylene glycols. As in the case of xylitol, this mechanism is particularly favored over bifunctional acid–base catalysts, where the basic sites specifically promote the retro-aldol fragmentation of sorbitol and its hexose intermediates and the metallic sites catalyze the subsequent hydrogenation of the fragmented aldehyde and ketone products. This controlled C–C scission via the retro-aldol route allows for maintaining high selectivity towards glycols, provided the reaction parameters and catalytic functionalities are appropriately balanced.

Sadier et al. [31] carefully studied the aqueous-phase hydrogenolysis sorbitol over Rh–ReO_x_/ZrO_2_ catalysts. The catalyst showed high hydrogenolysis activity, although a mixture of lineal (e.g., hexanepentaols, -tetraols, -triols, -diols, and hexanols) and cyclic C_6_ products was achieved at 473 K and 8.0 MPa H_2_. Interestingly, C–C bond cleavage was minimized by carefully controlling parameters. The initial reaction rate showed a zero-order dependence on sorbitol, indicating strong adsorption of polyols on the ReO_x_ species, potentially limiting access to sites that would otherwise promote C–C scission. The catalytic activity was found to follow the order Rh ≈ Pt > Ir, which was explained by the noble metal capacity for H_2_ dissociation and subsequent hydrogenation. The ReO_x_ species played a crucial role in promoting internal dehydration of sorbitol, leading to the formation of cyclic C_6_ compounds (e.g., sorbitans and isosorbide). As indicated in previous sections, this intramolecular dehydration competes directly with the desired C–O bond hydrogenolysis. High hydrogen pressure (e.g., 80 bar) was found to be crucial for favoring linear deoxygenated products by enhancing the C–O bond hydrogenolysis rate, which effectively suppressed the formation of cyclic ethers and minimized C–C bond cleavage. Conversely, lower pressures or higher temperatures disproportionately favor intramolecular dehydration, leading to increased yields of thermodynamically stable cyclic by-products. The sequential appearance of deoxygenated products (hexanepentaols, hexanetetraols, hexanetriols, hexanediols, and hexanols) confirmed multiple consecutive C–O bond cleavage pathways. Under optimized pressure and temperature conditions, C–C cleavage products were formed in minor amounts.

Jia et al. [68] developed an in-situ-formed PdZn alloy catalyst from Pd/TiO_2_ and ZnO for the selective hydrogenolysis of sorbitol. In combination with Mg_3_AlO_x_ as a solid base, the system achieved a 54.6% combined yield of 1,2-PD and EG at 473 K and 5.0 MPa H_2_. The formation and composition of the PdZn alloy were found to determine the activity and selectivity. Mechanistic studies identified sorbitol dehydrogenation as the rate-determining step. The reaction conditions and catalyst composition favored C–C cleavage, which resulted in low yields of deoxygenated hexanols (ca. 10%). In this system, the in-situ-formed PdZn alloy served as the active dehydrogenation site for sorbitol, producing hexose intermediates. The critical C–C bond cleavage step to yield C_2_ and C_3_ glycols was primarily catalyzed by the solid base Mg_3_AlO_x_. The synergy between the metal-catalyzed dehydrogenation and base-catalyzed retro-aldol condensation steps was key to achieving high selectivity. Kinetic isotope experiments confirmed that sorbitol dehydrogenation was the rate-determining step, implying that the formation of reactive intermediates through C–H bond activation dictates the overall reaction rate before retro-aldol cleavage occurs.

In an interesting study, Jin et al. [69] came up with a catalytic system able to perform sorbitol hydrogenolysis without external hydrogen or an added base. Thus, lattice-strained bimetallic PtPd nanoparticles supported on N-doped mesoporous carbon were used, while the absence of H_2_ and a base was covered by in situ hydrogen generation and multifunctional active sites derived from lattice strain and electronic interactions with the N-doped support, respectively. The system produced glycols with modest selectivity rates. This study is interesting in that it provides a base-free, H_2_-free strategy, paving the way for more sustainable and scalable routes for biomass polyol valorization. The unique performance of these bimetallic nanoparticles was due to their multifunctional nature, enabling sorbitol hydrogenolysis without external hydrogen or added base. This is achieved through in situ hydrogen generation (via C–H cleavage) and the presence of multifunctional active sites that influence C–C and C–O cleavage. The N-doped carbon support functioned as both a Lewis base, promoting the retro-aldol mechanism for C–C bond cleavage, and an electronic modulator. Lattice strain at the metal–N interface induced structural reconstruction in the PtPd nanoparticles (e.g., Pt lattice contraction and Pd phase segregation), altering their electronic properties and weakening metal–H bonding, which resulted in enhanced C–H, C–O, and C–C cleavage.

Liu et al. [70] demonstrated that hydrodeoxygenation of sorbitol, xylitol, and erythritol can be carried out while preserving the carbon backbone of the substrates over a Pt–WO_x_/SiO_2_ catalyst. Under optimized conditions (463 K), sorbitol yielded ca. 40% of hexanodiols, with minor amounts of C–C cracked products. The Pt–WO_x_ interface was identified as the active site responsible for regioselective C–O bond scission. The Pt–WO_x_ interface on the silica support was identified as the active site for regioselective C–O bond scission in sorbitol hydrodeoxygenation. While the catalyst efficiently preserved the carbon backbone, the tendency for C–O bond cleavage at specific positions (e.g., C_2_ secondary hydroxyl group) was attributed to the presence of WO_x_ species with strong Brønsted acidity. This suggested a direct C–O hydrogenolysis mechanism, distinguishing it from catalysts that primarily promote C–C retro-aldol fragmentation.

Conversely, the use of Ni as a metal resulted in complete selectivity to C–C cracked products [71]. Thus, La-promoted Ni/ZrO_2_ catalysts achieved nearly 100% sorbitol conversion with 74% combined selectivity to EG, 1,2-PD, and glycerol. The addition of La_2_O_3_ enhanced the Ni dispersion and surface basicity, promoting selective C–C bond cleavage via the retro-aldol pathway. A metal–alkali coordination mechanism was proposed to account for the high glycol selectivity. This enhanced glycol selectivity was attributed to a synergistic effect of enhanced Ni dispersion and increased surface basicity provided by the La_2_O_3_ promoter. This basicity specifically promoted selective C–C cleavage of sorbitol via the retro-aldol, leading to high yields of EG and 1,2-PD.

Zhu et al. [72] developed Ni/Mg_6_Al_4_O_x_ catalysts from NiMgAl–LDH precursors for sorbitol hydrogenolysis, achieving 97.9% conversion and 43.8% glycol yields at 523 K. The catalyst showed excellent hydrothermal stability and recyclability due to strong metal–support interactions, whereas conventional Ni/Al_2_O_3_ and Ni/MgO catalysts suffered from deactivation and leaching. DFT studies indicated that 1,2-PD was formed via glyceraldehyde from retro-aldol cleavage, identifying this as the rate-determining step of the overall reaction. DFT identified the formation of 1,2-PD from glyceraldehyde via retro-aldol cleavage as the rate-determining step. This highlights the crucial role of basic sites in facilitating the C–C bond scission of sorbitol and its intermediates, guiding the reaction towards C_3_ diols.

Biomass can also serve as a raw material for the production of carbon materials, which can then be used for sorbitol hydrogenolysis. In this sense, Chen et al. [73] developed a bimetallic Ru/WO_x_ catalyst supported on N-doped carbon derived from bamboo shoot biomass for the hydrogenolysis of sorbitol to glycols. The catalyst exhibited excellent activity in aqueous alkaline media, achieving 85% total selectivity to 1,2-PD and EG. Synergistic interactions between Ru and WO_x_ were found to improve hydrogen spillover and C–C bond cleavage via retro-aldol reactions. The catalytic performance was sensitive to the WO_x_ content, carbon carbonization temperature, and Ca(OH)_2_ loading. The biomass-derived, N-doped carbon support played a multifaceted role; it provided abundant anchoring sites via ligand effects with N by chemically coordinating with Ru and WOx, influencing their electronic properties and enhancing catalytic activity. Furthermore, N incorporation improved the catalyst dispersibility in aqueous media, which is crucial for efficient hydrogenolysis reactions. Ru favored dehydrogenation of sorbitol to form reactive ketohexose and aldohexose intermediates and was crucial to hydrogen activation, dissociating H_2_ molecules into atomic hydrogen that can spill over to the WO_x_ surface. This hydrogen spillover is significantly boosted by the WO_x_ modification, contributing to higher overall hydrogenolysis activity. Finally, WO_x_ species, particularly basic W^5+^ sites and associated oxygen vacancies, were found to be central to promote C–C bond cleavage process via retro-aldol condensation. These oxygen vacancies served as initial adsorption sites for sorbitol hydroxyl groups, favoring rapid dehydrogenation. Basic W^5+^ sites then promoted retro-aldol fragmentation of the ketohexose and aldohexose intermediates to C_2_ and C_3_ fragments (e.g., glycolaldehyde and dihydroxyacetone). Subsequently, the activated hydrogen atoms (from Ru, via spillover) rapidly hydrogenate these carbonyl intermediates to produce the target glycols.

When combined with solid bases, Ru/C yielded glycols with high selectivity [74]. Among them, CaZrO_x_ showed the best performance, reaching 50% selectivity to glycols with 74% sorbitol conversion (493 K, 4 h). The catalyst served as both as a depot for leached homogeneous Ca species and as a solid-phase promoter via synergistic effects of perovskite and ZrO_2_ phases. The performance of the catalyst decreased significantly until stabilizing in subsequent runs with 18% glycol selectivity. This bifunctionality facilitates the retro-aldol pathway for C–C bond cleavage in sorbitol hydrogenolysis, leading to high selectivity towards glycols. The interaction between the Ru metal and the solid basic support is key to enabling this selective fragmentation.

The same group [75] used N-doped activated carbons and achieved 80% selectivity to EG and 1,2-PD from sorbitol, while suppressing side reactions such as decarbonylation. The XPS analysis revealed that nitrogen on the support played an important role by withdrawing electrons from Ru, enhancing selectivity. The catalyst performance was consistent across various synthesis routes when Ru was loaded via equilibrium impregnation. These two studies highlight the importance of N-doped carbons as tunable supports for selective hydrogenolysis of sorbitol. The electronic modulation revealed by XPS enhanced the selectivity, likely by optimizing the interaction between the metal active sites and polyol intermediates to favor C–C bond cleavage via retro-aldol pathways over undesired side reactions such as decarbonylation. The tunability offered by N-doped carbons was found to be crucial for directing this selectivity.

Ru/C unpromoted catalysts were studied by Wang et al. [76]. The authors focused on the influence of Cu particle size and the Ca(OH)_2_ loading. The catalytic activity was found to increase with the particle size up to 14 nm, beyond which it plateaued. On the other hand, the product selectivity was unaffected by size but it was found to be highly dependent on the Ca(OH)_2_ concentration. An optimal 85% glycols selectivity rate was achieved at 503 K, 5 MPa H_2_, and 0.3 equiv. Ca(OH)_2_. Interestingly, the in-situ conversion of lactic acid to 1,2-PD contributed to the high selectivity. Catalyst deactivation was linked to Cu nanoparticle aggregation, suggesting a need for improved sintering resistance when using Cu. The reaction was governed by a complex interplay of the Cu particle size, concentration of basic promoter, and in situ conversion pathways. The overall mechanism involved dehydrogenation of the polyols on metal surfaces, the base-catalyzed retro-aldol condensation of the resulting sugar intermediates to form C_2_ and C_3_ fragments, and the rehydrogenation of these carbonyl intermediates to the target glycols. The effect of the Cu particle size on the catalyst performance was attributed to a higher fraction of catalytically active Cu(111) surface planes on larger nanoparticles, suggesting that specific crystallographic features are more active for dehydrogenation. However, since the selectivity remained constant with the particle size, the Cu particle size primarily dictated the reaction rate rather than the preference for C–C cleavage positions. The amount of Ca(OH)_2_ (base promoter) showed a profound impact on product selectivity by influencing the retro-aldol condensation. Ca(OH)_2_ was found to favor retro-aldol cleavage of ketose intermediates over aldose intermediates (yielding C_2_ and C_4_ fragments). Lactic acid and glycerol by-products were in-situ-converted to 1,2-PD during the reaction. This in situ valorization contributed significantly to the remarkably high overall glycol selectivity. Catalyst deactivation was attributed to the sintering of Cu nanoparticles, as revealed by TEM.

Alexzmand et al. [77] synthesized Cr_2_O_3_–SiO_2_ catalysts via sol–gel methods and evaluated them for sorbitol hydrogenolysis in aqueous media. A 20 wt% Cr_2_O_3_-loaded catalyst showed the best performance, achieving 70.1% sorbitol conversion and up to 60% glycol yields under optimized conditions. High acidity and uniform mesoporosity contributed to high catalytic activity, as result of synergistic Cr–Si interactions. The 20CrSi catalyst demonstrated good reusability over five cycles with minimal loss in performance. These findings highlight 20CrSi as a promising, stable catalyst for green glycol production from biomass. The synergistic Cr–Si interactions likely play a role in promoting the selective C–C bond cleavage pathways, including retro-aldol condensation, leading to high glycol yields under optimized conditions.

It is necessary to consider that retro-aldol condensations are important pathways during hydrodeoxygenation of sorbitol. The cleavage of C–C bonds through retro-aldol reactions yields C_4_ and C_3_ intermediates such as erythrose, glyceraldehyde, and glycolaldehyde, which subsequently undergo hydrogenolysis to produce short-chain glycols such as EG and propylene glycol. This pathway is particularly favored over bifunctional acid–base catalysts, where basic sites facilitate retro-aldol fragmentation while metal sites catalyze subsequent hydrogenation. The retro-aldol route represents a controlled fragmentation strategy that maintains high selectivity toward glycols when reaction parameters and catalytic functionalities are properly balanced [71,72,73,74,75,76,77]. Thus, tailoring the acid–base properties of the support remains essential to directing the pathway and maximizing yields toward desired glycol products.

Isosorbide, a dehydrated derivative of sorbitol, was selectively hydrogenolyzed by Chen et al. [78] using a heterogeneous Rh/SiO_2_ catalyst. The system achieved a 58% total yield of C_6_ diols and triols, outperforming analogous reactions with glucose and sorbitol. The catalyst enabled C–O bond cleavage without significant C–C scission, preserving the carbon framework. Interestingly, significant amounts of 1,6-hexanediol, a highly valuable diol, were obtained.

**Figure 7:**
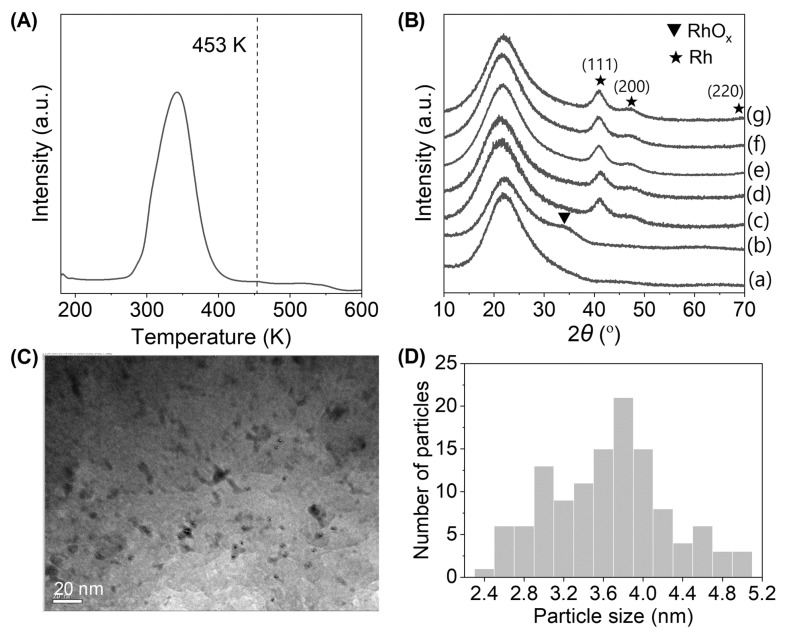
Characterization data for the Rh/SiO_2_ catalyst: (**A**) TPR profile; (**B**) XRD patterns tracking changes in the catalyst from calcination through different reaction times (0, 1, 4, 6, and 8 h); (**C**) TEM image of the catalyst after 4 h of reaction; (**D**) distribution of Rh particle sizes [78].

The Rh/SiO_2_ catalyst was thoroughly characterized using hydrogen temperature-programmed reduction (H_2_-TPR), XRD, and TEM. The TPR analysis (Figure 7A) confirmed that the Rh species were fully reduced to their metallic state below 453 K, reaching their active form under reaction conditions. XRD patterns (Figure 7B) further supported these results, showing a broad RhO_x_ signal after calcination that completely transformed into distinct metallic Rh peaks (at 41, 47, and 70°) after reaction. No RhO_x_ signals were observed post-reaction. TEM results (Figure 7C,D) showed no significant aggregation of the Rh particles after reaction, suggesting good dispersion and stability of the metallic Rh phase under hydrothermal conditions. The Rh/SiO_2_ demonstrated good reusability and stability over multiple cycles. These results position isosorbide as a promising intermediate for polyol synthesis via Rh-catalyzed hydrogenolysis.

**Table 3:** Summary of recent catalytic systems for the selective hydrogenolysis of sorbitol.

Catalyst	Conditions	Main Product Selectivity (%)	Reference
Rh–ReO_x_/ZrO_2_	473 K, 8.0 MPa H_2_; batch reactor; 5 wt % of sorbitol in water; 0.5 g catalyst	55 (1,4-sorbitan)	[79]
Pd/TiO_2_ +ZnO + Mg_3_AlO_x_	493 K, 5.0 MPa H_2_; batch reactor; 10 wt % of sorbitol in water; 1 g catalyst; reaction time 4 h	42 (1,2-PG)	[68]
Pt–Pd (lattice-strained core–shell)	473 K, 1 MPa N_2_; batch reactor; 0.3 kmol/m^3^ of sorbitol in water; 0.1 g catalyst; reaction time 8.5 h	43 (glycols)	[69]
Pt/WO_x_–SiO_2_	463 K, 8.0 MPa H_2_; batch reactor; W/Pt = 0.25, molar ratio	65 (C_6_ triols and diols)	[70]
Rh/SiO_2_	453 K, 8.0 MPa H_2_, batch reactor; isosorbide: 4 g in 2 g H_2_O, 0.6 g catalyst, 4 h.	58 (C_5_ and C_6_ diols and triols)	[78]
Ni/La_2_O_3_/ZrO_2_	493 K, 4.0 MPa H_2_; batch reactor; 10% sorbitol; catalyst amount = 1 g; 4 h.	60 (EG + 1,2-PD)	[71]
Ni/Mg_6_Al_4_Ox	523 K, 6.0 MPa H_2_; sorbitol 4 g in 40 mL of water; 0.2 g of catalyst; 6 h	44 (EG + 1,2-PD)	[72]
Ru/WO_x_ supported on N-doped carbon	473 K, 4.0 MPa H_2_; batch reactor; 2 g sorbitol in 20 mL of water, 0.1 g catalyst, 0.2 g Ca(OH)_2_; 6 h	85 (EG + 1,2-PD)	[73]
Ru/C in combination with mixed metal oxides of alkaline earth metals and Ti or Zr	473 K, 8.0 MPa H_2_; batch reactor; 0.5 g sorbitol in 10 mL of water, 0.025 g of 5 %Ru/C and 0.225 g of mixed metal oxide; 4 h	50 (1,2-PD)	[74]
Ru on N-doped Carbon	473 K, 8.0 MPa H_2_; batch reactor; 0.2 g catalyst; 0.3 g Ca(OH)_2_, 20 mL of H_2_O; 4 h	80 (EG + 1,2-PD)	[75]
Cu/AC	513 K, 5.0 MPa H_2_; batch reactor; 0.18 M sorbitol, 0.08 g Cu, 0.2 g Ca(OH)_2_, 20 ml H_2_O; 25 h.	84 (EG + 1,2-PD)	[76]
Cr_2_O_3_-SiO_2_	493 K, 3.0 MPa H_2_; batch reactor; 10 wt% of aqueous sorbitol solution; 0.4 g of catalyst; 2 h	60 (EG + 1,2-PD)	[77]

## 4. Scalability Challenges and Continuous-Flow Systems

Most studies on selective hydrogenolysis of biomass polyols have been conducted in batch reactors at the milliliter scale. While batch operation is suitable for mechanistic insights and catalyst screening, it suffers from non-uniform heat and mass transfer, variability, and difficulty in extrapolating the catalyst lifetime under hydrothermal conditions to process-relevant timescales. These constraints complicate technoeconomic analyses and provide performance metrics sensitive to transient concentration profiles [33]. When it comes to industrial deployment, industrial biorefineries require stable operation in a steady state with well-defined residence time distributions, reliable heat removal, and continuous product withdrawal, which are more naturally achieved in continuous-flow configurations [9,25,33]. Fixed-bed flow operation can, in principle, stabilize selectivity by continuously removing diols from acidic or metallic sites before secondary dehydration, retro-aldol cleavage, or hydrogenolysis to lighter products are carried out. Several attempts have been made recently to carry out selective C–O scission of diols under continuous flow operation. In a vapor-phase fixed-bed study, Kantam et al. [44] reported that a Pt–WO_x_/SBA-15 catalyst achieved 86% glycerol conversion with 42% selectivity to 1,3-PD at 483 K and 1 atm, with a carbon balance above 98%. Time-on-stream tests at a WHSV of ca. 1 h^−1^ revealed stable performance and allowed the authors to identify deactivation pathways linked to WO_x_ restructuring. In a liquid-phase continuous flow system, Wang et al. [54] demonstrated that WO_x_-supported Pt catalysts operated at 413 K and 1.0 MPa H_2_ with 5 wt% glycerol sustained a 1,3-PD space–time yield of 3.78 g gPt^−1^ h^−1^. Importantly, no detectable Pt agglomeration was observed after 75 h on stream, underscoring the hydrothermal stability of the interfacial Pt–WO_x_ sites. Complementarily, Luo et al. [55] tested egg shell Ir–ReO_x_/SiO_2_ pellets in a trickle-bed reactor, achieving 61% glycerol conversion with 31% selectivity to 1,3-PD at 403 K and 8 MPa H_2_ (WHSV = 0.5 h^−1^) after 36 h of continuous operation. The egg shell distribution was found to minimize diffusion limitations and enabled preferential scission of secondary hydroxyl groups.

While glycerol has been the primary focus of continuous-flow hydrogenolysis studies, reports on higher polyols such as xylitol, sorbitol, or mannitol (C_5_–C_6_) remain very rare in the literature. The main limitation is their very low volatility, which complicates vapor-phase operation, the most common configuration in fixed-bed continuous reactors. In liquid-phase systems, their high viscosity and strong hydrogen-bonding interactions further complicate feed handling, lead to mass transfer limitations, and accelerate catalyst deactivation through coke formation and salt deposition. As a result, as shown in Table 2, the vast majority of C_5_–C_6_ polyol hydrogenolysis studies continue to rely on batch reactors, despite their limited scalability. Overcoming these constraints will require reactor engineering strategies tailored to non-volatile, viscous feeds, such as slurry-phase continuous stirred tank designs, structured trickle beds, or coupling with in situ extraction and membrane separation. These strategies could include process intensification combining reaction and separation. For example, as stressed by Ai et al. [42], biphasic extraction can partition products away from catalytic sites and attenuate condensation and cyclization, thereby preserving carbon efficiency during hydrogenolysis. Likewise, the effect of the solvent nature on the hydrogenolysis selectivity has been well documented for Cu–ZnO systems, thereby providing a guide for the choice of co-solvents or co-feeds in flow to modulate intermediate solvation and inhibit over-reactions [46].

## 5. Overcoming Barriers in Selective Hydrogenolysis of Polyols to Diols

Despite the significant advancements in the catalytic selective hydrogenolysis of biomass-derived polyols into diols described in previous sections, several critical barriers persist, hindering the commercial viability and widespread use of these technologies. Addressing these challenges is crucial for unlocking the full potential of biomass as a sustainable source for chemicals. The main barrier lies in the inherent chemical complexity and high reactivity of the biomass polyols, which typically results in modest yields towards the desired polyols. Thus, a multitude of undesirable byproducts, including shorter diols, cyclic ethers (e.g., isosorbide), oligomers, or C–C cleavage products, are typically found by researchers. Rational catalyst design is, therefore, required to direct the synthesis towards the targeted diols. Thus, bifunctional catalysts with precisely tuned metal–acid site proximity and strength allow fine control over the regioselectivity of the hydrogenolysis step via tandem dehydration and hydrogenation fast steps. In addition, the use of reducible oxide supports (e.g., TiO_2_, Nb_2_O_5_, CeO_2_) enabling SMSI in combination with oxophilic promoters (e.g., Re, W, Mo, Sn) can activate specific secondary C–O bonds to direct selectivity towards targeted diols. Intramolecular cyclization remains an important issue, particularly over acid catalysts. A judicious selection of solvent polarity can be a proper way to circumvent internal cyclodehydration of biomass polyols. Alternatively, the inherent tendency of some polyols to cyclize can be strategically exploited by developing catalysts designed to subsequently cleave these cyclic intermediates (e.g., isosorbide) into linear diols, transforming a potential hindrance into a valuable pathway, as recently exploited by Chen et al. [78].

A second significant barrier deals with catalyst stability and longevity, particularly under the harsh hydrothermal conditions (i.e., high temperatures and pressures in an aqueous environment) typically employed for polyol hydrogenolysis. Under these severe conditions, heterogeneous catalysts, especially those containing noble metals, are prone to undergo deactivation, leaching, sintering, and coke deposition issues, limiting the catalyst’s lifetime and increasing potential operational costs. To enhance the catalyst’s robustness, research efforts should concentrate on developing new supports with superior hydrothermal stability and resistance to leaching, such as N-doped carbon materials [75], which are particularly known for allowing strong metal–support attachments in SACs. Designing catalysts with tailored pore structures can minimize byproduct formation that leads to coke deposition, which combined with the utilization of in situ hydrogen-forming agents (e.g., formic acid) can mitigate deactivation. Catalyst deactivation is particularly relevant when dealing with crude feedstocks. For instance, while most mechanistic and catalytic studies rely on purified model polyols (e.g., glycerol, xylitol, sorbitol), industrial implementation will require tolerance to more complex feedstocks such as crude glycerol from biodiesel production and polyol mixtures from lignocellulosic hydrolysates. Crude glycerol, generated at multi-million-ton scales as a byproduct of transesterification, typically contains 40–80 wt% glycerol along with methanol, soaps, salts, and free fatty acids. These impurities can poison active sites, promote coke formation, or alter acid–base equilibria, thereby lowering the catalyst’s lifetime and selectivity. Strategies under investigation include pre-treatments (e.g., neutralization, esterification), the design of robust bifunctional catalysts resistant to alkali metals and organics, and process integration schemes that valorize co-present compounds rather than removing them. Similarly, lignocellulosic hydrolysates contain a mixture of C_5_ and C_6_ polyols along with sugars, furans, and organic acids. Their compositional variability complicates selective hydrogenolysis, as competing reactions such as dehydration, condensation, or over-hydrogenation can dominate. Emerging research shows that tuning catalyst acid–base properties, employing membrane or biphasic extraction systems, and leveraging size-selective porous supports can help maintain selectivity to target diols under these more challenging conditions. Broadening the substrate scope from model compounds to crude and mixed feedstocks, thus, represents a critical step toward the commercial viability of bio-based diol production.

Metal leaching has been reported in several noble metal–oxide systems. Ir–ReO_x_/TiO_2_ catalysts for glycerol hydrogenolysis were found to undergo partial dissolution of Re species during aqueous operation, which progressively diminished the bifunctional synergy responsible for secondary C–O bond scission [43,55]. Similarly, WO_x_ domains in Pt–WO_x_/Al_2_O_3_ were shown to leach under acidic conditions, destabilizing the acid–metal interface required for 1,3-PD formation [53,54]. In Ni-based formulations for sorbitol hydrogenolysis, an inductively coupled plasma (ICP) analysis confirmed gradual metal loss to the liquid phase, further highlighting the generality of leaching across both noble and base metal catalysts [71,72]. Nanoparticle sintering and restructuring are particularly critical for noble metals. TEM and operando X-ray absorption spectroscopy (XAS) of Pt–WO_x_ catalysts revealed dynamic migration and agglomeration of Pt nanoparticles during long-term runs, leading to a loss of isolated Pt–WO_x_ interfacial sites essential for high 1,3-PD selectivity [44,55,59,60]. In Ru systems, particle size effects were directly linked to selectivity shifts between C–O and C–C cleavage, with larger Ru domains favoring over-hydrogenolysis pathways [45]. Studies on PdZn alloy catalysts under sorbitol hydrogenolysis demonstrated in situ restructuring into active PdZn phases, with subsequent destabilization during extended operation, pointing to the dual role of alloy dynamics in activity and deactivation [68]. PtPd nanocatalysts further illustrated this issue, since lattice strain initially promoted transfer hydrogenolysis activity but relaxed under reaction, modifying the electronic structure and active site ensembles [69]. Likewise, Pt–WO_x_/SiO_2_ underwent support-assisted restructuring, as observed by environmental TEM, causing Pt redispersion and WO_x_ aggregation under hydrothermal stress [70]. Coking and carbonaceous deposition remain ubiquitous causes of deactivation during polyol hydrogenolysis. Operando infrared (IR) spectroscopy of NiMo_2_C/Al_2_O_3_ and Ni/ZrO_2_ catalysts demonstrated progressive accumulation of strongly bound glyceroxide intermediates, which blocked metal sites and lowered diol selectivity [47,71]. For sorbitol hydrogenolysis, coke formation was found to scale with the polyol chain length, with Rh–ReO_x_/ZrO_2_ and Ru–WO_x_/C catalysts particularly susceptible due to over-condensation of intermediates [73,79]. Recent studies on hydrothermally stable Ni- and La-promoted Ni/ZrO_2_ confirmed that while structural integrity could be maintained, carbon buildup on active sites was the dominant pathway to performance decay [72,74]. Even in noble metal systems, such as Rh/SiO_2_ for isosorbide conversion, carbonaceous deposits accumulated during extended runs and were clearly correlated with declining yields [78]. These mechanistic insights are enabled by advanced characterization techniques. Thus, in situ XAS provided quantitative evidence of Pt oxidation state oscillations and metal–support coordination changes under hydrogenolysis conditions [53,54,59]. Environmental TEM captured nanoparticle migration, coalescence, and WO_x_ restructuring in real time [60,70]. Operando diffuse reflectance infrared Fourier transform spectroscopy (DRIFTS) and attenuated total reflectance infrared spectroscopy (ATR-IR) allowed direct detection of adsorbed intermediates and carbonaceous residues, linking coke accumulation with reduced selectivity [47,71,72,73]. Together, these studies demonstrate that stability issues are not secondary to selectivity design but are intimately tied to the atomic-scale structure of bifunctional sites.

A recurring challenge in the selective hydrogenolysis of biomass polyols is the control of C–C bond cleavage. While selective C–O scission yields target diols such as 1,3-PD, 1,2-PD, and EG, uncontrolled C–C fragmentation decreases carbon efficiency by producing lighter glycols or even methane, thereby decreasing process sustainability. The balance between C–O and C–C pathways is determined by the interplay of metal dispersion, alloying, support acidity, and reaction environment. Metal particle size effects provide direct evidence for C–C selectivity control. For Ru catalysts, increasing the particle size favored over-hydrogenolysis, where larger domains enhanced C–C scission relative to C–O cleavage [45]. Similar size-dependent behavior was observed in Pt–WO_x_ catalysts, where small Pt clusters at the WO_x_ interface promoted selective dehydration–hydrogenation sequences, while larger Pt aggregates enhanced parallel C–C fragmentation [53,59,60]. Bifunctional acid–metal systems further highlight this competition. On Rh–ReO_x_/ZrO_2_, C–C bond cleavage increased with the polyol chain length, consistent with retro-aldol condensation routes that become more favorable for C_5_–C_6_ sugar alcohols [79]. WO_x_-modified Pt/SiO_2_ showed that stable interfacial acid sites can suppress retro-aldol fragmentation, thereby preserving high selectivity to C_4_–C_6_ diols [70]. These findings indicate that tuning the acid strength and proximity to metal centers is essential to disfavor C–C scission. Alloying strategies also play a decisive role. PdZn alloys formed in situ during sorbitol hydrogenolysis demonstrated reduced C–C cleavage compared with monometallic Pd, as alloying modified the electron density and adsorption geometry of glyceroxide intermediates [68]. PtPd nanocatalysts revealed the double-edged role of lattice strain; although the activity was initially enhanced, electronic distortion simultaneously promoted side C–C cleavage channels [69]. Catalyst instability and coking are tightly linked to C–C cleavage selectivity. In NiMo_2_C/Al_2_O_3_ and Ni/ZrO_2_ catalysts, operando infrared spectroscopy detected strongly adsorbed glyceroxide species that progressively condensed, leading to coke buildup and subsequent retro-aldol fragmentation [47,71,72]. For Ru–WO_x_ supported on N-doped carbon, coking accelerated the transition from selective C–O scission to uncontrolled fragmentation, revealing how deactivation processes can directly modulate cleavage pathways [73]. Likewise, in sorbitol hydrogenolysis over solid base catalysts, deactivation via condensation reactions was directly associated with carbon balance losses [74]. In noble metal systems such as Rh/SiO_2_ applied to isosorbide hydrogenolysis, coke deposition was similarly linked to C–C fragmentation and lower diol yields [78].

The third barrier deals with the use of expensive noble metals. Thus, many of the most effective catalytic systems for polyol hydrogenolysis currently employ costly noble metals (e.g., Pt, Rh, Ir, Ru), which constitutes a substantial portion of the overall process cost and impedes large-scale implementation. Future efforts must intensify the development of highly active and selective non-noble metal catalysts (e.g., Cu, Ni, Co, Fe, Sn, Mo) and their bimetallic combinations or alloys. Ideally, these alternative materials should provide synergistic effects in order to potentially achieve comparable or even superior performance as compared to their noble metal counterparts. Maximizing the catalyst turnover frequency (TOF) and overall catalyst efficiency is also critical to reduce the required catalyst loading and reduce scale up costs.

The underexplored nature of various polyol substrates and the inherent variability of feedstocks represent additional barriers for the implementation of polyol deoxygenation technologies. As described in previous sections, significant progress has been achieved with model polyols such as glycerol, xylitol, and sorbitol, while the hydrogenolysis of less-studied polyols (e.g., mannitol, arabitol), and more importantly complex polyol mixtures derived directly from lignocellulosic hydrolysates, remains largely unexplored. This feedstock variability poses considerable challenges for maintaining consistent product quality and process efficiency in biorefineries. Future research must broaden the substrate scope to encompass a wider array of biomass-derived polyols and their mixtures, leading to the development of more versatile catalytic systems.

The fifth barrier involves the challenges of process intensification and successful scale-up. The vast majority of current research studies are conducted at the laboratory scale, and translating these findings to industrial-scale operations presents substantial hurdles with regards to reactor design, efficient heat and mass transfer, complex product separation, and the establishment of robust continuous operation. To overcome these scaling challenges, there is a strong need to prioritize the development and optimization of continuous-flow reactor systems (e.g., fixed-bed reactors), since the vast majority of studies operate with batch reactors. Additionally, designing integrated cascade reaction systems that combine hydrogenolysis with other catalytic steps can lead to more efficient production and separation of target diols. The judicious utilization of advanced computational tools and artificial intelligence (AI) for accelerated catalyst discovery and optimization will also be crucial in enabling more rational design of catalysts and reaction conditions tailored for industrial applications, as recently shown by Kang and co-workers [80]. Finally, on a fundamental level, a deeper mechanistic understanding of reaction pathways and intermediates remains essential for rational catalyst development. Operando spectroscopy and theoretical modeling (e.g., DFT, microkinetic simulations) are increasingly employed to understand site requirements for selective bond activation, as recently demonstrated by Zhu et al. [72].

Finally, some important polyols such as erythritol and mannitol remain significantly unexplored as a source of diols. Erythritol (meso-1,2,3,4-butanetetraol) is an emerging C_4_ platform chemical with significant potential for diol production. Its transformation into value-added diols, particularly butanediols, has received attention. Erythritol deoxygenation to diols has been mostly attempted by hydrodeoxygenation and deoxydehydration [81]. Hydrodeoxygenation over Ir-ReO_x_/SiO_2_ resulted in selective C–O bond dissociation, yielding 1,4-butanediol (e.g., up to 25% yield) but with potential for over-reaction to 1-butanol at longer times. Rh-ReO_x_ catalysts also promote C–O hydrogenolysis, producing mixtures of mixtures of butanediol isomers. Mechanistic insights suggest that reducible metal oxides (e.g., ReO_x_) modify noble metal (Ir, Rh) activity, influencing H_2_ activation and substrate binding. Additionally, 1,4-anhydroerythritol (1,4-AHERY), a erythritol dehydration product, has been explored as a feedstock with catalysts such as Rh-MoO_x_/SiO_2_, yielding 2-butanol with modest yields. A comprehensive review of the recent literature reveals a noticeable scarcity of dedicated studies focusing on the selective transformation of mannitol into diols via catalytic hydrodeoxygenation or similar processes. Despite mannitol’s relevance as a biomass-derived polyol, recent publications detailing its efficient conversion to specific diols within the scope of this review are not prominently available.

Although this review focuses on heterogeneous catalytic hydrogenolysis, it is worth noting that enzymatic and microbial routes to diols have also been explored [82,83,84]. For instance, glycerol can be transformed into 1,3-PD by engineered microorganisms expressing glycerol dehydratase and 1,3-PD oxidoreductase, while xylitol and sorbitol are routinely produced enzymatically from lignocellulosic sugars. Enzyme-catalyzed conversion of these polyols into diols such as 1,2-PD has been reported, albeit with limitations in productivity and stability. These examples confirm the feasibility of biocatalytic routes, yet current industrial practice remains dominated by thermocatalysis due to its superior scalability and robustness.

## 6. Conclusions

This review examines the significant steps made in the selective deoxygenation of biomass-derived polyols into valuable diols. Focusing exclusively on recent advances, it highlights the remarkable developments in the hydrogenolysis of key polyols such as glycerol, erythritol, xylitol, and sorbitol. The development of sophisticated bifunctional catalysts, often integrating noble metals with oxophilic promoters and engineered supports, has been crucial to allow selective hydrogenolysis. These innovations have led to unprecedented control over C–O bond cleavage, resulting in enhanced selectivity towards target diols such as EG, 1,2-PD and 1,3-PD. The improved understanding of reaction mechanisms, influenced by synergistic metal–acid interactions and the catalyst structure, continues to drive the rational design of more efficient and selective catalytic systems for a sustainable bio-based chemical industry.

Despite these encouraging developments, several critical barriers persist, hindering the widespread commercialization and industrial application of selective polyol hydrogenolysis technologies. The inherent chemical complexity of biomass polyols often results in a challenging array of undesired byproducts, necessitating highly precise catalyst design. Furthermore, issues related to catalyst stability and longevity under demanding hydrothermal reaction conditions remain a significant concern. The prevalent reliance on costly noble metals in many high-performing systems represents an economic hurdle and stresses the need for active and selective non-noble metal alternatives with significantly increased turnover frequencies. Addressing the underexplored nature of less-studied polyols and complex polyol mixtures directly derived from lignocellulosic hydrolysates also poses a substantial feedstock variability challenge.

Continued research must prioritize overcoming these identified barriers to fully unlock the potential of biomass-derived diols. Key future directions include expanding the substrate scope to effectively process diverse lignocellulosic hydrolysates, which will require the development of more versatile and tolerant catalysts. Efforts must be made toward process intensification and successful scale-up, moving from batch laboratory-scale investigations to continuous-flow systems for industrial feasibility. The strategic integration of advanced computational tools and AI for accelerated catalyst discovery and optimization will be instrumental in enabling more rational and predictive design of catalysts and reaction conditions tailored for industrial applications. Such advancements, coupled with a deeper mechanistic understanding, will ultimately pave the way for transformative and sustainable diol production from renewable biomass resources.

## Figures and Tables

**Table 2:** Representative studies on the selective hydrogenolysis of xylitol to glycols. Reaction conditions include the temperature, hydrogen pressure, and solvent system; product selectivity rates are reported as percentages to EG and 1,2-PD under the specified conditions.

Catalyst System	Reaction Conditions	Selectivity to PDs	Reference
Ru/C	423–463 K, 3–5 MPa H_2_, H_2_O, alkaline promoters	EG (40–50%), 1,2-PD (30–40%)	[61]
Ru/C + KOH or MnO	433–473 K, 4 MPa H_2_, aqueous phase	EG (35–45%), 1,2-PD (25–30%)	[62]
Ni/Ce–TiO_2_ (mesoporous)	493 K, 3 MPa H_2_, H_2_O	EG (45–55%), 1,2-PD (25–35%)	[63]
Pd–Pt/TiO_2_	453 K, ~1 MPa H_2_ (in situ from formic acid), H_2_O	EG (30–35%), 1,2-PD (40–45%)	[64]
Cu–Ni–ZrO_2_	493 K, 5 MPa H_2_, H_2_O, base-free	EG (40–50%), 1,2-PD (30–35%)	[65]
Ni–Cu/SiO_2_	473–493 K, 4 MPa H_2_, H_2_O	EG (45–55%), 1,2-PD (25–35%)	[66]
Ru/MnO/C	453–493 K, 4 MPa H_2_, H_2_O, no base	EG (50%), 1,2-PD (30%)	[67]
Ni/C, Ni/C + basic promoters	473–493 K, 5 MPa H_2_, H_2_O	EG (40–45%), 1,2-PD (35–40%)	[31]
